# Thermoforming Vacuum Packaging Influences Fresh Pork Loin Chop Characteristics

**DOI:** 10.3390/foods13172701

**Published:** 2024-08-27

**Authors:** Brooks W. Nichols, Gabriela M. Bernardez-Morales, Savannah L. Douglas, Gabriella F. Johnson, Ricardo J. Barrazueta-Cordero, Aeriel D. Belk, Jase J. Ball, Jason T. Sawyer

**Affiliations:** Department of Animal Sciences, Auburn University, Auburn, AL 36849, USA; bwn0004@auburn.edu (B.W.N.); gzb0063@auburn.edu (G.M.B.-M.); sld0060@auburn.edu (S.L.D.); gfj0006@auburn.edu (G.F.J.); rzb0143@auburn.edu (R.J.B.-C.); adb0097@auburn.edu (A.D.B.); jase.ball@zoetis.com (J.J.B.)

**Keywords:** instrumental color, overwrap, pork, shelf life, vacuum packaging

## Abstract

The storage duration of fresh meat products is a contributing factor leading to increased waste and loss at the retail counter. Losses of fresh pork can be linked to packaging methods that do not protect the attributes of color, taste, and odors consumers use in determining wholesome meat. Boneless pork loins (N = 63) were fabricated into 2.54-cm-thick chops and assigned to one of three vacuum treatments (VacA, VacB, VacC) or a fourth polyvinyl chloride overwrap (PVC) treatment to assess objective fresh color, cook loss, Warner–Bratzler shear force (WBSF), and lipid oxidation. Pork chops (n = 882) were evaluated at 5-day intervals (D 0, 5, 10, 15) in a randomized complete block design. Pork chop surface color was lighter (L*; *p* < 0.0001) when stored in a vacuum compared to PVC-packaged loin chops, regardless of storage duration. Redness (a*) values were greater (*p* < 0.0001) for loin chops stored in PVC than all other vacuum packaging treatments throughout the entire 15-day display period. Relative values for chroma on PVC-packaged loin chops were greater (*p* < 0.0001) throughout the simulated retail display period. An interaction of day and packaging treatment (*p* < 0.0343) occurred for WBSF. Lipid oxidation for pork chops packaged using PVC was significantly greater (*p* < 0.0001) from Day 10 through the completion of the storage period. Results indicate that vacuum packaging limits the deterioration of fresh pork loin chops, whereas traditional overwrapping expedites the color and lipid oxidation during refrigerated storage.

## 1. Introduction

Packaging for meat and food products is continuously evolving to meet both product quality and consumer satisfaction demands. At the retail level, consumer perception of meat quality is based primarily on surface color as an indicator of safe and wholesome meat [1]. Unfortunately, over storage time, the surface color of meat products may change, and packaging methods may alter the consumers’ indicator of freshness. Changes occurring during retail storage may cause a visual appraisal to be a less accurate predictor of fresh meat quality. Consumer purchasing decisions are heavily biased by surface color, and products perceived to be discolored will require discounting or discarding by retailers regardless of product wholesomeness and quality [2]. Identifying the impact of packaging materials that will protect meat quality characteristics during retail storage is needed when current retail storage of fresh meat is often limited to less than 7 days in the U.S.

Food losses within the meat, poultry, and fish industries account for almost USD 48 billion in lost revenue annually [3]. Meat packaging is a tool that can aid in reducing these revenue losses by extending storage and reducing throwaways that retail outlets incur. Factors attributing to the greater percentage of meat wastage are often caused by poor packaging, deterioration of visual characteristics, and consumer perceptions of quality within meat products [4]. Challenges within the meat industry often include efforts to reduce meat waste or loss at retail, but identifying improved packaging methods and fresh or frozen storage conditions may result in a solution to the financial losses that occur.

Global pork consumption has increased over the past two decades and is projected to continue increasing through 2030 [5,6]. With pork consumption projected to increase, identifying methodologies for extending the storage duration of pork products without altering quality is essential. Previous research has concluded that vacuum packaging pork and colder storage temperatures have been successful in extending the storage duration of some fresh pork products [7]. However, packaging film technology is changing, and limited results are available, highlighting the uses of thermoforming vacuum packaging. Research has also identified some alternative packaging methods, such as vacuum skin packaging, that alter lipid oxidation and stabilize the surface color of fresh beef and pork products [8,9,10]. Furthermore, extended storage of fresh pork using vacuum packaging with traditional pouches has improved objective tenderness when compared to products stored in oxygen-rich overwrapping for at least 14 days [11,12]. Understanding the improvements in color stability and objective tenderness during extended storage by vacuum packaging could benefit the pork industry by creating a more preferred consumer product. Therefore, more information regarding packaging methods is needed to measure meat quality changes that occur during storage periods.

It is not well known how the composition of thermoforming vacuum packaging films used during meat packaging may alter the storage of meat products. Thermoforming packaging films are designed in a multitude of variations to either reduce or increase oxygen transmission rate which may prove effective in storing fresh pork products longer [13]. The objectives of the current study were to evaluate thermoformed vacuum packaging and polyvinyl chloride (PVC) overwrapping packaging methods on the quality attributes of fresh pork loin chops during a 15-day retail storage period.

## 2. Materials and Methods

### 2.1. Sample Preparation

Whole boneless pork loins were purchased from a commercial retailer six days after slaughter and transported to the Lambert Powell Meat Laboratory at Auburn University (Auburn, AL, USA). Pork loins were stored in original vacuum shrink packaging (Winpak Ltd., Winnipeg, MB, Canada) in a refrigerated (2.0 ± 1.3 °C) room (Model LEH0630, Larkin, Stone Mountain, GA, USA) for nine days in the absence of light to simulate logistical transportation from harvest to a case-ready manufacturer as used throughout the U.S. meat industry. After storage, boneless pork loins (N = 63) were removed from packaging and fabricated fresh using a boneless cutting blade fixed to a BIRO bandsaw (Model 334, Biro Manufacturing Company, Marblehead, OH, USA) into 2.54-cm-thick chops (N = 882 chops; n = 14 chops/loin). Pork loins were divided into quarters (n = 4 chops/quarter) and quarters assigned to one of four packaging treatments. Packaged chops were individually identified and randomly assigned to instrumental surface color, cook loss, lipid oxidation, and Warner–Bratzler shear force.

### 2.2. Packaging Treatments

Vacuum-packaged pork loin chops (n = 220/treatment) were packaged individually using a Variovac Optimus system (OL0924, Variovac, Zarrentin am Schaalsee, Germany). Loin chops were randomly assigned to one of three thermoforming vacuum packaging films, VacA, VacB, VacC, and sealed with a standardized non-forming film constructed with the following parameters: 75 μ nylon/EVOH/enhanced/polyolefin plastomer coextrusion with an oxygen transmission rate of 0.10 cc/sq. m/24 h and a vapor transmission rate of 4.0 g/sq. m/24 h. Thermoformed vacuum packages were formed with 0.800 bar of heat at 108.1 ± 1.4 °C and 0.650 bar of pressure. The sealing die was reduced to 5 bar of vacuum and sealed at 135.0 ± 1.1 °C. PVC overwrapped packaged chops were placed on a foam tray (1S, GENPAK, Charlotte, NC, USA) with an absorbance pad (DRI-LOC AC-50, Novipax, Oak Brook, IL, USA) and hand wrapped with a PVC film (O_2_ transmission rate = 14,000 cc O_2_/m/24 h/atm). Film composition and oxygen transmission rates of vacuum films are displayed below (Table 1).

### 2.3. Simulated Retail Display Conditions

After packaging, all cut and packaged pork loin chops (N = 882) were placed in an LED-lit, 3-tier display case (Model TOM-60DX-BN, Turbo Air Inc., Long Beach, CA, USA) operating at 3.0 ± 1.2 °C to simulate fresh retail display conditions during a 15-day storage period. Chops were displayed under constant lighting 2297 lux (TOM-600-12-V4-3, Phillips Xitanium, Seoul, Republic of Korea) throughout the 15-day display period. Data loggers (ThermoData WiFi Loggers, Thermoworks, American Fork, UT, USA) were used to monitor the temperatures inside the retail cases for the duration of the study. Packaged chops were rotated in the display case daily to simulate consumers moving products in a retail setting. Objective measurements for all chops occurred on Days 0, 5, 10, and 15 of the simulated storage.

### 2.4. Instrumental Color

Pork loin chops (n = 42/treatment) were assessed for instrumental surface color with a HunterLab MiniScan EZ colorimeter, Model 45/0 LAV (Hunter Associates Laboratory Inc., Reston, WV, USA). Prior to instrumental color readings, the colorimeter was standardized using black and white tiles through each of the packaging films per the manufacturer’s guidelines for instrument calibration. Three readings were captured through the packaging film of each chop using illuminant D65, and an average of the readings was calculated. Pork chops were evaluated for surface color lightness (L*), redness (a*), and yellowness (b*). Hue angle (HA) and Chroma (CHMA) were calculated from the L*, a*, and b* values using the equations √a*^2^ + b*^2^ and tan^−1^(b*/a*), respectively. Red to brown (RTB) was calculated using the measured reflectance ratio of 630 nm:580 nm and is indicative of the color shift from red to brown. Relative myoglobin percentage values were calculated as deoxymyoglobin (%DMb = {2.375 × [1 − ({A473 − A700})]} × 100), metmyoglobin (%MMb = {[1.395 − ({A572 − A700}/{A525 − A700})]} × 100), and oxymyoglobin (%OMb = 100 − (%MMb + %DMb)). References for calculations of objective color readings were adopted from the American Meat Science Association for color measurement [14].

### 2.5. Cook Loss and Warner–Bratzler Shear Force

Prior to cooking, loin chops were removed from individual packaging (n = 35/treatment/day), and excess surface moisture was blotted dry with a paper towel. Chops were weighed on an analytical balance (PB3002-S, Mettler Toledo, Greifensee, Switzerland) and then cooked in a convection oven (Vulcan, Baltimore, MD, USA) preheated to 192.2 °C until an internal temperature of 70.0 °C. Cooked chops were removed from the oven, cooled to room temperature (24.2 °C), and re-weighed so cook loss could be calculated using the following equation: % Cook Loss = ((Raw Wt. (g) − Cooked Wt. (g)) ÷ Raw Wt. (g)) × 100. After weighing, six 1.27 cm diameter cores were removed from each chop by a handheld corer parallel to the muscle fiber and sheared once perpendicular to the muscle fiber [15]. Objective tenderness was measured using a texture analyzer (Model Ta-XT Plus100C, Texture Technologies Corp., South Hamilton, MA, USA) equipped with a 50 kg load cell and a crosshead speed of 50 mm/min. Maximum peak force values for each core were averaged to calculate the average peak force (N) value.

### 2.6. Lipid Oxidation

Lipid oxidation of pork chops was evaluated by 2-thiobarbituric acid reactive substances (TBARS). Chops (n = 10/treatment/day) were removed from individual packaging, trimmed of subcutaneous fat, and cut into approximate 2.0 ± 0.05 g samples. Samples were homogenized (Model PT 10-35, Brinkman Inst., Westbury, NY, USA) for 30 s with 8 mL of cold 50 mM phosphate buffer (pH 7.0) and 2 mL of trichloroacetic acid (VWR Chemicals, Solon, OH, USA). After homogenizing, the homogenate was filtered through Whatman No. 1 filter paper, and the filtrate was separated into duplicate 2 mL aliquots. Aliquots were combined with 2 mL of 0.02 M 2-thiobarbituric acid reagent (MP Biomedicals, Solon, OH, USA) and cooked in a water bath (100.1 °C) for 20 min. After cooking, sample tubes were placed directly into an ice bath for 15 min. Absorbance was measured using a spectrophotometer (Model UV-1600PC, VWR International LLC, Randor, PA, USA) set at 533 nm. Absorbance readings were multiplied by 12.21, and values were recorded as mg malondialdehyde (MDA)/kg of fresh meat as previously described [16].

### 2.7. Statistical Analysis

Data were analyzed as a randomized complete block design using the GLIMMIX procedures of SAS (version 9.2; SAS Inst., Cary, NC, USA). Treatment and day were fixed effects, with loin as a blocking variable and replication as the random effect when calculating values for cooking losses, objective tenderness, and lipid oxidation. For instrumental color values, the day was included in the model as a random effect. Orthogonal contrasts were computed for comparing vacuum packaging treatments to the PVC treatment (VacA, VacB, and VacC vs. PVC overwrapped). Least square means were computed for all variables, and significant (*p* ≤ 0.05) means were separated using pairwise *t*-tests (PDIFF option).

## 3. Results and Discussion

### 3.1. Instrumental Color

An interactive (*p* < 0.0001) effect of treatment × day occurred for objectively measured lightness (L*) of fresh pork chops (Table 2). Vacuum-packaged chops were objectively lighter (*p* < 0.0001) than PVC-packaged chops on Days 10 and 15 of the storage period. However, the objective color of PVC-packaged chops became darker (*p* < 0.0001) as storage time increased. Orthogonal contrasts calculated for objective surface lightness confirm that packaging treatments of vacuum and PVC chops differed (*p* = 0.0074), indicating that vacuum packaging resulted in a lighter surface color than PVC packaging. Surface lightness values of pork chops in all vacuum packaging films were 1.8% lighter than chops packaged in PVC film and likely caused by the interaction with atmospheric gases during storage. Packaging materials for fresh meat have barrier properties to reduce atmospheric gases from interacting with color proteins such as myoglobin. In the current study, pork chops stored in a vacuum remained lighter and less susceptible to darkening throughout the entire storage period. Changes in surface color lightness have been observed and documented throughout the literature for fresh and frozen meat cuts. The oxygen transmission rate of packaging films has not been evaluated extensively when used for packaging fresh meat products such as pork. Improvements in vacuum packaging technologies, such as the use of thermoforming films may afford greater storage periods of fresh meats such as pork in the retail counter within the U.S.

Current results agree with previous research that vacuum packaging meat can protect against deterioration of L* values better than meat packaged using materials with greater exposure to atmospheric gases [17,18,19]. It is plausible that the increased surface reflectance measured using objective color methods for vacuum-packaged chops may be caused by pork muscle variation in pH, as reported in previous research [8]. Nevertheless, the mean pH values of all pork loins (n = 63) in this study were 5.71 and likely not the source of surface lightness variation that was measured objectively in either PVC or thermoforming vacuum packaging of fresh pork chops. Many factors are responsible for surface color deterioration, including pH, and have been well documented throughout the literature, including the age of raw material, packaging materials, storage temperature, duration of storage, microbial organisms, and exposure to atmospheric gases. Additional research is needed to quantify the growth of spoilage organisms that occur in fresh pork when using thermoforming packaging.

In addition to changes in the objective lightness of the pork chop surface, an interaction (*p* < 0.0001) of packaging treatment × day occurred for redness (a*) during the 15-day retail display period (Table 2). Loin chops packaged in PVC were redder (*p* < 0.0001) than chops packaged in all vacuum treatments throughout the entire storage period. Orthogonal contrasts calculated for redness confirm (*p* < 0.0001) packaging method (PVC vs. VAC) can influence the surface redness of fresh pork chops and was likely caused by the ability of oxygen to pass more freely through the PVC packaging film and bind with myoglobin on the chopping surface. Pork chops packaged in PVC were 63% redder than chops packaged in vacuum. A lack of redness on pork loin chops under vacuum suggests that the OTR of the vacuum film should be altered to allow for greater passage of oxygen through the film layers and binding with the meat surface to alter surface color during storage. Most notably, proteins with greater red fibers can appear redder under vacuum [20]. A lack of redness in vacuum-packaged pork chops observed in the current study does not agree with previous literature on vacuum-packaged ground beef in which redness values were not altered during a 10-day storage period [20]. Conversely, a* values do support the conclusions of previous literature, reporting redness of vacuum-packaged beef and pork will decrease after day zero and not deteriorate during storage periods [21,22]. Changes over time in meat surface redness will occur; however, the speed at which those changes occur varies among packaging methods such as PVC, vacuum, and modified atmosphere. Consumers rely heavily on surface color for purchase decisions, and the many factors that affect surface redness, such as packaging film, atmosphere, and myoglobin quantity, remain a priority for investigation to limit the decline of surface color during storage [12].

There was no interaction for packaging treatment × day (*p* = 0.8118) of objective yellowness (b*) values (Table 2). Orthogonal contrasts for pork chop yellowness indicate that packaging can cause variations in objective yellow surface color (*p* < 0.0001) when storing fresh pork in vacuum and PVC methods. Packaged chops in PVC films had 15.5% more yellow surface color than vacuum-packaged chops. Current results do not agree with previous literature highlighting decreasing b* values of loin chops when stored under vacuum, causing the surface to remain more yellow than green throughout the respective display period [23,24]. It has been documented that pork products packaged in vacuum can have lower b* values than products packaged using oxygen-rich methods such as PVC [21].

Relative values for spectral color across the wavelengths of 400 to 700 nm resulted in an interaction of treatment × day for hue angle (*p* < 0.0001), chroma (*p* < 0.0001), and red to brown (*p* < 0.0001). Throughout the storage period, pork loin chops packaged in PVC had less (*p* < 0.0001) hue angle shifting from red to yellow and were closer to the true red than chops packaged using vacuum treatments (Table 3). Additionally, chop surface vividness (CHMA) was greater (*p* < 0.0001) in PVC overwrapped chops than in chops packaged using a vacuum (Table 3). Red to brown (RTB) values were greatest (*p* < 0.0001) for PVC-packaged chops on day 0 of the storage period, but the packaging did not alter RTB values at the conclusion of the storage period (Table 3). Orthogonal contrasts calculated for HA, CHMA, and RTB spectral values confirm that differences observed in surface color variation were a result of the packaging treatment (*p* ≤ 0.0001). Vacuum-packaged chops were 12.3% farther from the true red axis than PVC-packaged chops, suggesting packaging films blocked the surface interaction of myoglobin with atmospheric gases. Additionally, CHMA values were 21.2% more vivid for PVC-packaged chops than vacuum-packaged chops, supporting a brighter, more vivid color when oxygen can interact with myoglobin on the meat surface. However, pork chops stored in PVC packages had only 4.5% calculated redder surfaces to a change in brown. Greater hue angle values observed in the current study for vacuum-packaged pork chops differ from the results of previous research reporting no difference in hue angle values of pork steaks regardless of packaging method [25]. Moreover, chroma values in the current study differ from those of previous research, where chroma values in vacuum-packaged ground beef remain unchanged during storage [20]. Results of the current study suggest relative spectral values for vacuum-packaged loin chops will have limited surface color redness and vividness throughout storage. A lack of surface color may be detrimental to the sale of fresh pork cuts at the retail counter when consumers have historically placed great emphasis on surface color. However, additional research is necessary to identify the mechanisms of color changes that occur in pork when using vacuum packaging. Limitations throughout the documented literature are focused more on meat proteins such as beef steaks and grind with a greater reaction between oxygen and myoglobin.

A treatment × day interaction occurred for calculated myoglobin values of deoxymyoglobin (*p* < 0.0001), oxymyoglobin (*p* < 0.0001), and metmyoglobin (*p* < 0.0001). Relative values of deoxymyoglobin for PVC-packaged chops were the least (*p* < 0.0001) on Day 0 of storage and greatest for VacC on Day 10 (Table 4). Surface color in PVC chops became darker as storage time increased, and the surface color deterioration likely caused the calculated values to increase. Greater relative calculations of deoxymyoglobin for vacuum-packaged pork chops in the current study agree with previous results that vacuum-packaged pork will appear to have a darker surface color [21]. Additionally, oxidation of myoglobin can occur more rapidly in PVC-packaged meat, supporting the resulting increase in relative deoxymyoglobin values of PVC loin chops at Day 10 [26]. An interaction of packaging and treatment resulted in the greatest (*p* < 0.0001) relative values of oxymyoglobin on Day 0 for PVC-packaged chops (Table 4). Packaging can be instrumental in protecting the surface color changes that occur during storage. Current results indicate vacuum packaging is a method that can hinder the deterioration of surface color changes, whereas PVC often results in a rapid rate of color change. Lastly, metmyoglobin values were greatest (*p* < 0.0001) for PVC chops on Day 5 and Day 10 but the least (*p* = 0.0002) for VacC packaged chops on Day 5, indicating that myoglobin had oxidized more rapidly on chops when using PVC packaging films. Barrier properties of packaging films can reduce partial pressures within the package and limit the exposure of the meat surface to oxygen. Noted in the current study, VacC film had the lowest oxygen transmission rate, and PVC had the greatest. Orthogonal contrasts indicate that packaging treatments of vacuum and PVC differed (*p* < 0.0001) for calculated relative forms of myoglobin, with PVC-packaged chops having greater (*p* < 0.0001) values of oxymyoglobin and metmyoglobin while vacuum-packaged chops had greater (*p* < 0.0001) values of deoxymyoglobin than PVC. Pork chops stored in PVC packaging objectively appeared to have 73.2% MET and 9.3% OXY, whereas chops in vacuum had 73.9% more DEO. Percent differences of objectively measured surface color values suggest that packaging film does alter the surface color of fresh meats. It has been reported previously that metmyoglobin values of beef steaks packaged in PVC are greater after 10 days of storage supporting the results of the current study for PVC-packaged chops [27]. Contrary to current results, previous literature reports that an increase in MET values of vacuum-packaged beef patties can occur when stored in refrigerated conditions [18]. It is plausible that the decrease in MET values observed after Day 5 is the result of myoglobin succumbing to oxidation. However, it is important to note that vacuum packaging of pork chops afforded more surface color protection over the 15-day storage than PVC packaging.

### 3.2. Cook Loss and Warner–Bratzler Shear Force

Objective measures of tenderness and moisture loss throughout refrigerated storage can assist with identifying possible impacts consumers may highlight after retail purchases and consumption. A treatment × day interaction (*p* ≤ 0.0040) occurred for the percentage cook loss of pork loin chops (Table 5). Cooking loss was greater (*p* < 0.0001) for pork chops packaged in VacA from day 0 through day 10 of the storage period and least (*p* < 0.0001) for chops packaged in PVC on Day 10 (Table 5). It is plausible that differences in cooking loss can be attributed to the protective nature of the vacuum packaging film reducing evaporative moisture losses during storage and subsequent greater concentrations of free moisture to lose during cooking. Orthogonal contrasts indicate packaging method can influence moisture losses during storage, causing greater losses during cooking in chops packaged using a thermoforming vacuum compared to PVC-packaged chops. Objective tenderness for pork chops may be trivial, but differences in objective tenderness of 0.04% for PVC stored chops suggest more research is needed to ascertain the influence of packaging on the tenderness of whole-muscle pork chops. Current results suggest that barrier protection because of vacuum packaging limits evaporation. Therefore, greater moisture was available to lose during cooking, ultimately causing tenderness values to increase. Regardless, additional research is needed to evaluate the mechanism of packaging influences on both subjective and objective measures of tenderness and cookery.

In addition to the cooking loss interaction, there was also a treatment × day interaction (*p* = 0.0343) for objective tenderness (WBSF) that occurred during storage conditions (Table 5). Objective tenderness values were greatest (*p* = 0.0010) for VacA packaged chops on Day 0 compared to all other packaging treatments and least (*p* = 0.0074) on Day 10 for chops packaged in PVC. Orthogonal contrasts calculated for WBSF confirm that packaging treatments of vacuum and PVC differed (*p* ≤ 0.0207) and likely caused an increase in objective tenderness through moisture losses that occurred during the cooking phase. Cooking losses of pork chops stored in thermoforming packaging were 22% greater than pork chops in PVC packaging. It is likely that the chops stored in oxygen-permeable packaging had greater evaporation of free moisture during storage, leading to less moisture losses during cooking. Results of the current study support previous literature reporting that vacuum-packaged pork chops can have greater WBSF values than pork packaged in the presence of greater oxygen concentrations [11,12]. Differences in WBSF values in the current study do not agree with the results of previous literature reporting that pork packaged aerobically had greater WBSF values than pork packaged in a vacuum [28]. Objective tenderness values in meat can be impacted by factors such as packaging, as reported in the current study, but other factors have also been documented to alter tenderness, such as protein linking, cookery method, degree of doneness, and oxidation, which were not analyzed in this study. Nevertheless, additional research is necessary to illustrate the influence packaging imparts on objective tenderness measurements of pork chops following refrigerated storage.

### 3.3. Lipid Oxidation

There was a treatment × day interaction (*p* < 0.0001) for lipid oxidation evaluated using 2-thiobarbituric acid reactive substances (TBARS) throughout the simulated storage period (Table 6). Lipid oxidation values were greatest (*p* < 0.0001) in PVC-packaged chops on Day 15 compared to pork chops stored using thermoforming vacuum packaging. TBARS values were two times greater with the use of PVC at the conclusion of the storage period than with all chops stored using thermoforming vacuum packaging. Orthogonal contrasts calculated were significant (*p* < 0.0001) and indicate that vacuum packaging properties, which reduce oxygen and vapor transmission, can hinder lipid oxidation during storage compared to PVC. Pork chops stored in PVC packaging resulted in 35% more lipid oxidation than vacuum-packaged stored pork chops, suggesting that barrier films can reduce the interaction with atmospheric oxygen, causing deterioration. Lipid oxidation in oxygen-permeable films (PVC) increased; however, all TBARS values for loin chops in all vacuum-packaged treatments were well below the detectable threshold by consumers of 0.5 mg malonaldehyde. It has been reported that TBARS values greater than 0.5 mg MDA/kg are linked to the creation of off-flavors during consumer sensory evaluation, and TBARS values of loin chops packaged in PVC exceeded this threshold at Day 10 [8]. Greater TBAR values of PVC-packaged chops in the current study on Day 15 support the results of previous literature reporting PVC-packaged pork fillets having greater TBAR values compared to vacuum-packaged samples [8]. Changes in TBARS values for chops packaged in vacuum agree with previous literature which concluded TBARS values did not change, but TBARS did and can increase for pork packaged when the presence of oxygen is greater [9,21]. In the current study, loin chops packaged in PVC had TBARS values that would reflect potential sensory off-flavors from Day 10 through the completion of the display period, while chops packaged in vacuum did not meet the 0.5 level at any point during storage. It is plausible that the storage period needed to be increased to reflect a greater increase of lipid oxidation when using vacuum packaging. However, the delayed oxidation of lipids when using thermoforming vacuum packaging is promising information for retail pork products that may need extended storage.

## 4. Conclusions

Results indicate that oxygen permeability and vapor transmission of thermoforming packaging films do alter objective surface color, specifically the redness of fresh pork chops during retail storage. PVC packaging of fresh pork chops can cause greater oxidation of myoglobin altering surface color and even lipid deterioration. Surprisingly, vacuum packaging increased cooking loss and even objective tenderness when compared to an oxygen-permeable packaging method such as PVC. Thermoforming vacuum packaging is a method that can reduce meat losses or waste on the retail counter by stabilizing surface color and lipid deterioration. Furthermore, vacuum packaging affords consumers the avenue to recycle packaging materials than traditional PVC overwrapped meat packaging that is sent to landfills. It is apparent throughout the current investigation that additional research is needed to fully understand the surface color changes that occur when storing pork retail cuts and subjective flavor attributes when using thermoforming vacuum packaging films.

## Figures and Tables

**Table 1:** Manufacturer specifications for packaging films used during fresh retail storage of pork loin chops.

Treatment ^1^	Composition	Thickness	Puncture	OTR ^2^	VPR ^3^
VacA	polyethylene/EVOH/polyethylene coextrusion	150 μ	25 N	0.6 mL/m/ 24 h	3.2 g/m /24 h
VacB	polyethylene/EVOH/polyethylene coextrusion	175 μ	28 N	0.5 mL/m/ 24 h	2.8 g/m /24 h
VacC	polyethylene/EVOH/polyethylene coextrusion	200 μ	31 N	0.4 mL/m/ 24 h	2.4 g/m /24 h
PVC	Polyvinyl chloride	10 μ	--	14,000 mL/m/24 h	30 g/m/24 h

^1^ Packaging treatment defined as (VacA, VacB, VacC, PVC). ^2^ OTR: Oxygen transmission rates. ^3^ VPR: Vapor transmission rates.

**Table 2:** Objective surface color (L*, a*, b*) values of pork loin chops during retail storage.

		Packaging Treatment ^1^					*p*-Value		
Day	VacA	VacB	VacC	PVC	SEM ^2^	Contrast ^3^	Treatment	Day	Treatment × Day
L*									
0	57.44 ^def^	56.72 ^ef^	57.35 ^def^	57.46 ^def^	0.494	0.0074	0.0543	<0.0001	<0.0001
5	58.02 ^bcde^	57.43 ^def^	57.60 ^cdef^	57.88 ^cde^					
10	58.71 ^abcd^	58.37 ^abcd^	58.19 ^bcd^	56.41 ^f^					
15	59.62 ^a^	58.91 ^abc^	59.30 ^ab^	56.38 ^f^					
a*									
0	3.96 ^d^	4.03 ^d^	3.87 ^de^	8.72 ^a^	0.190	<0.0001	<0.0001	<0.0001	<0.0001
5	3.13 ^f^	3.42 ^ef^	3.28 ^f^	7.03 ^b^					
10	2.93 ^fg^	2.94 ^fg^	2.91 ^fg^	4.60 ^c^					
15	2.62 ^g^	2.94 ^fg^	3.02 ^fg^	4.68 ^c^					
b*									
0	12.78	11.95	12.11	14.24	0.136	<0.0001	<0.0001	<0.0001	0.8118
5	11.75	11.52	11.52	13.66					
10	11.86	11.78	11.69	13.62					
15	11.92	11.85	11.82	13.72					

^1^ Packaging Treatment: VacA (150 μ polyethylene/EVOH/polyethylene coextrusion), VacB (175 μ polyethylene/EVOH/polyethylene coextrusion), VacC (200 μ polyethylene/EVOH/polyethylene coextrusion), and PVC (polyvinyl chloride overwrap). ^2^ SEM, Standard error of the mean for the interaction of treatment × day. ^3^ Orthogonal Contrast VAC (VacA + VacB + VacC) vs. PVC. ^a–g^ Mean values lacking common superscripts within a color measurement differ significantly (*p* < 0.05).

**Table 3:** Interactive effect of packaging × storage day on calculated spectral values of pork loin chop.

	Packaging Treatment ^1^						*p*-Value		
Day	VacA	VacB	VacC	PVC	SEM ^2^	Contrast ^3^	Treatment	Day	Treatment × Day
HA ^4^									
0	71.94 ^de^	71.29 ^e^	72.20 ^de^	58.60 ^g^	0.843	< 0.0001	<0.0001	<0.0001	<0.0001
5	75.03 ^bc^	73.47 ^cd^	74.01 ^bcd^	63.00 ^f^					
10	76.12 ^ab^	75.96 ^ab^	75.93 ^ab^	71.12 ^e^					
15	77.59 ^a^	76.05 ^ab^	75.51 ^abc^	71.01 ^e^					
CHMA ^5^									
0	12.86 ^d^	12.70 ^d^	12.79 ^d^	16.76 ^a^	0.135	<0.0001	<0.0001	<0.0001	<0.0001
5	12.23 ^e^	12.13 ^e^	12.07 ^e^	15.46 ^b^					
10	12.29 ^e^	12.25 ^e^	12.15 ^e^	14.46 ^c^					
15	12.27 ^e^	12.31 ^e^	12.29 ^e^	14.56 ^c^					
RTB ^6^									
0	1.88 ^d^	1.92 ^cd^	1.91 ^d^	2.41 ^a^	0.029	0.0001	0.0009	<0.0001	<0.0001
5	1.93 ^bcd^	2.00 ^b^	2.00 ^b^	1.89 ^d^					
10	1.90 ^d^	1.95 ^bcd^	1.99 ^bc^	1.92 ^cd^					
15	1.87 ^d^	1.93 ^bcd^	1.90 ^d^	1.89 ^d^					

^1^ Packaging Treatments: VacA (150 μ polyethylene/EVOH/polyethylene coextrusion), VacB (175 μ polyethylene/EVOH/polyethylene coextrusion), VacC (200 μ polyethylene/EVOH/polyethylene coextrusion), and PVC (polyvinyl chloride overwrap). ^2^ SEM, Standard error of the mean for the interaction of treatment × day. ^3^ Orthogonal Contrast VAC (VacA +VacB + VacC) vs. PVC. ^4^ HA: Hue angle represents the change from true red (greater value indicates a greater shift from red to yellow). ^5^ CHMA: Chroma, a measure of total color (a larger number indicates a more vivid color). ^6^ RTB: Red to brown, calculated as 630 nm reflectance/580 nm reflectance representing a change in surface color from red to brown (larger value indicates a redder color). ^a–g^ Means lacking a common superscript within a spectral value differ significantly (*p* < 0.05).

**Table 4:** Interactive effect of packaging × storage day on relative myoglobin values of fresh pork loin chops during retail storage.

	Packaging Treatment ^1^						*p*-Value		
Day	VacA	VacB	VacC	PVC	SEM ^2^	Contrast ^3^	Treatment	Day	Treatment × Day
DEO ^4^									
0	27.24 ^e^	27.64 ^de^	28.63 ^d^	6.23 ^h^	0.429	<0.0001	<0.0001	<0.0001	<0.0001
5	33.14 ^ab^	32.74 ^abc^	32.71 ^bc^	7.28 ^g^					
10	32.97 ^abc^	33.17 ^ab^	33.91 ^a^	22.19 ^f^					
15	32.03 ^bc^	31.90 ^c^	32.05 ^bc^	22.35 ^f^					
OXY ^5^									
0	57.81 ^e^	56.51 ^ef^	57.21 ^e^	78.08 ^a^	0.688	<0.0001	<0.0001	<0.0001	<0.0001
5	56.21 ^ef^	61.35 ^cd^	64.37 ^b^	64.99 ^b^					
10	55.25 ^fg^	56.76 ^ef^	58.00 ^e^	53.98 ^g^					
15	57.16 ^e^	60.69 ^d^	63.11 ^bc^	60.70 ^d^					
MET ^6^									
0	14.95 ^cd^	15.75 ^cd^	14.17 ^d^	15.69 ^cd^	0.78	<0.0001	<0.0001	<0.0001	<0.0001
5	10.65 ^e^	5.92 ^hi^	2.93 ^j^	27.73 ^a^					
10	11.79 ^e^	10.07 ^ef^	8.09 ^fg^	23.83 ^b^					
15	10.82 ^e^	7.41 ^gh^	4.84 ^ij^	16.96 ^c^					

^1^ Packaging Treatments: VacA (150 μ polyethylene/EVOH/polyethylene coextrusion), VacB (175 μ polyethylene/EVOH/polyethylene coextrusion), VacC (200 μ polyethylene/EVOH/polyethylene coextrusion), and PVC (polyvinyl chloride overwrap). ^2^ SEM, Standard error of the mean for the interaction of treatment × day. ^3^ Orthogonal Contrast VAC (VacA + VacB + VacC) vs. PVC. ^4^ DEO, relative values of deoxymyoglobin using spectral values. ^5^ OXY, relative values of oxymyoglobin using spectral values. ^6^ MET, relative values of metmyoglobin using spectral values. ^a–j^ Means within a myoglobin state lacking a common superscript differ significantly (*p* < 0.05).

**Table 5:** Interactive effect of packaging × storage day on cooking loss and objective tenderness values of fresh pork loin chops.

	Packaging Treatment ^1^				*p*-Value				
Day	VacA	VacB	VacC	PVC	SEM ^2^	Contrast ^3^	Treatment	Day	Treatment × Day
CL% ^4^									
0	33.83 ^a^	29.86 ^abc^	26.89 ^cdef^	25.18 ^ef^	1.453	<0.0001	<0.0001	0.0103	0.004
5	33.82 ^a^	31.99 ^ab^	25.60 ^def^	24.97 ^efg^					
10	33.77 ^a^	28.52 ^bcde^	27.17 ^cdef^	21.10 ^g^					
15	26.07 ^cdef^	24.03 ^fg^	29.46 ^bcd^	24.55 ^efg^					
WBSF ^5^									
0	27.95 ^a^	20.76 ^bcd^	19.56 ^d^	20.44 ^cd^	1.526	0.0207	<0.0001	0.0933	0.0343
5	24.45 ^abc^	19.55 ^d^	17.69 ^d^	20.71 ^bcd^					
10	24.62 ^abc^	19.13 ^d^	21.06 ^bcd^	18.73 ^d^					
15	24.83 ^ab^	19.77 ^d^	26.73 ^a^	20.64 ^bcd^					

^1^ Packaging Treatments: VacA (150 μ polyethylene/EVOH/polyethylene coextrusion), VacB (175 μ polyethylene/EVOH/polyethylene coextrusion), VacC (200 μ polyethylene/EVOH/polyethylene coextrusion), and PVC (polyvinyl chloride overwrap). ^2^ SEM, Standard error of the mean for the interaction of treatment × day. ^3^ Orthogonal Contrast VAC (VacA + VacB + VacC) vs. PVC. ^4^ CL%: Percent cook loss. ^5^ WBSF: Warner–Bratzler Shear Force, evaluated in Newtons (N). ^a–g^ Means lacking a common superscript differ significantly (*p* < 0.05).

**Table 6:** Interactive effect of packaging × storage day on TBAR values of pork loin chops.

	Packaging Treatment ^1^					*p*-Value			
Day	VacA	VacB	VacC	PVC	SEM ^2^	Contrast ^3^	Treatment	Day	Treatment × Day
0	0.34 ^cde^	0.36 ^cd^	0.31 ^e^	0.33 ^de^	0.016	<0.0001	<0.0001	<0.0001	<0.0001
5	0.33 ^de^	0.34 ^cde^	0.33 ^de^	0.37 ^cd^					
10	0.35 ^cde^	0.37 ^cd^	0.33 ^de^	0.55 ^b^					
15	0.38 ^c^	0.33 ^de^	0.33 ^cde^	0.71 ^a^					

TBAR: 2-thiobarbituric acid reactive substances, reported as mg/kg of malonaldehyde in fresh tissue. A larger value indicates greater oxidation. ^1^ Packaging Treatments: VacA (150 μ polyethylene/EVOH/polyethylene coextrusion), VacB (175 μ polyethylene/EVOH/polyethylene coextrusion), VacC (200 μ polyethylene/EVOH/polyethylene coextrusion), and PVC (polyvinyl chloride overwrap). ^2^ SEM, Standard error of the mean for the interaction of treatment × day. ^3^ Orthogonal Contrast VAC (VacA + VacB + VacC) vs. PVC. ^a–e^ Means lacking a common superscript differ significantly (*p* < 0.05).

## Data Availability

The original contributions presented in the study are included in the article, further inquiries can be directed to the corresponding author.
